# Evaluating oseltamivir prescriptions in Centers for Medicare and Medicaid Services medical claims records as an indicator of seasonal influenza in the United States

**DOI:** 10.1111/irv.12552

**Published:** 2018-03-25

**Authors:** F. Scott Dahlgren, David K. Shay, Hector S. Izurieta, Richard A. Forshee, Michael Wernecke, Yoganand Chillarige, Yun Lu, Jeffrey A. Kelman, Carrie Reed

**Affiliations:** ^1^ Centers for Disease Control and Prevention National Center for Immunization and Respiratory Diseases Influenza Division Atlanta GA USA; ^2^ Center for Biologics Evaluation and Research Food and Drug Administration Silver Spring MD USA; ^3^ Acumen LLC. Burlingame CA USA; ^4^ Centers for Medicare and Medicaid Services Washington DC USA

**Keywords:** antivirals, influenza, medicare, surveillance, validity

## Abstract

**Background:**

Over 34 million residents of the United States aged 65 years and older are also Medicare prescription drug beneficiaries. Medical claims records for this age group potentially provide a wealth of data for better understanding influenza epidemiology.

**Objective:**

The purpose of this study was to evaluate data on oseltamivir dispensing extracted from medical claims records as an indicator of influenza activity in the United States for the 2010‐11 through 2014‐15 influenza seasons.

**Methods:**

We used Centers for Medicare and Medicaid Services (CMS) medical claims data to evaluate the weekly number of therapeutic oseltamivir prescriptions dispensed following a rapid influenza diagnostic test among beneficiaries 65 years old and older as an indicator of influenza timing and intensity. We compared the temporal changes in this indicator to changes in the proportion of influenza‐like illnesses among outpatient visits in the US Outpatient Influenza‐like Illness Surveillance Network (ILINet) by administrative regions defined by the US Department of Health and Human Services. Using the moving epidemic method, we determined intensity thresholds and categorized the severity of seasons for both CMS and ILINet data.

**Results:**

Centers for Medicare and Medicaid Services oseltamivir data and ILINet data were strongly correlated by administrative region (median Spearman's ρ = 0.78; interquartile range = 0.73‐0.80). CMS oseltamivir data and ILINet data substantially agreed (Cohen's weighted κ = 0.62) as to the seasonal severity across administrative regions.

**Conclusions:**

Our results support the use of oseltamivir dispensing in medical claims data as an indicator of US influenza activity.

## 1 INTRODUCTION

National and international surveillance for influenza informs important decisions, such as selecting vaccine components.^1^ In the United States, national surveillance for influenza has multiple components including syndromic surveillance for influenza‐like illness (ILI) in an outpatient setting and laboratory‐based surveillance for detecting influenza viruses from clinical specimens.^2^ Other components of national surveillance include inpatient sentinel surveillance for laboratory‐confirmed influenza, surveillance for deaths attributable to pneumonia and influenza, and surveillance for influenza‐associated pediatric mortality.^2^, ^3^, ^4^ Additional data sources—such as medical records, survey data, and Internet search queries—improve context when interpreting statistics from national surveillance.^5^

Medical claims data provide valuable insight into care provided to patients covered by the payer. Previous research found a strong correlation between national surveillance for ILI in the United States and prescriptions for antivirals with activity against influenza.^6^ Medicare claims data are particularly attractive for analysis of influenza in the United States, as patients 65 years old and older are a large population at risk of influenza infection, and over 34 million people in this age group are Medicare prescription drug beneficiaries.^7^, ^8^ However, these data only include information needed for billing and do not include results from diagnostic assays. Previous research captured trends in ILI surveillance at national and regional levels using syndromic data on outpatient visit diagnosis codes in medical claims data, but these results could represent illnesses from a number of respiratory pathogens.^9^ While developing an outcome to detect influenza‐associated clinic visits in a study assessing the relative effectiveness of high‐dose vs standard‐dose influenza vaccines, we noted temporal associations between Medicare claims for oseltamivir prescriptions and national surveillance for influenza viruses in clinical specimens.^10^ Here, we investigate whether trends in oseltamivir prescriptions dispensed to Medicare beneficiaries are temporally and spatially associated with trends in outpatient ILI and trends in detection of influenza viruses in clinical specimens

## 2 METHODS

The Centers for Medicare and Medicaid Services (CMS) compiles billing claims for services rendered to beneficiaries by healthcare providers. We linked claims data for Medicare Part B and Part D, which pay for community‐level care and prescription drugs, respectively. Community‐level care includes physician services and other non‐inpatient services such as durable medical equipment, laboratory services, and imaging services. From January 3, 2010, until October 31, 2015, we compiled both the weekly number of therapeutic oseltamivir prescriptions of 75 mg twice daily for 5 days among beneficiaries 65 years old and older and the subset dispensed within 2 days of a rapid influenza diagnostic test (RIDT). Therefore, we excluded prophylactic prescriptions of oseltamivir at a once daily rate. We used oseltamivir dispensed within 2 days of a RIDT because this suggests the clinician knew the result of the RIDT when prescribing oseltamivir.^11^ We stratified the data by administrative region as defined by the Department of Health and Human Services. We defined week 1 as the first week with at least 4 days in a year. Each influenza season started on week 40 and ended the following year on week 39. We evaluated the weekly number of oseltamivir prescriptions—with and without a RIDT—as a potential indicator of influenza activity.

To assess seasonal severity, we applied the moving epidemic method (MEM) developed by Vega and others.^12^ These researchers applied the MEM to data for ILI and acute respiratory illnesses in 28 European countries to demonstrate the method's value in normalizing disparate data.^13^ Additionally, the researchers applied the MEM to regional surveillance networks within Spain to demonstrate the method's value in early detection of influenza.^14^ One component of the MEM defines epidemic periods for each season, an interval when influenza activity is high. Another component of the MEM is constructing intensity thresholds (ITs) using confidence intervals. We applied the MEM to our data as follows: For each region, we found the 6 largest weekly counts from each of the 5 epidemic periods from the 2010‐11 through 2014‐15 seasons. For each region, we used these 30 counts to construct 1‐sided 100% × (1 ‐ α) confidence intervals for the geometric mean assuming a lognormal distribution at α = 0.50, 0.10, and 0.02. We labeled the upper limits of these confidence intervals as IT_50_, IT_90_, and IT_98_. We categorized each season as a low‐severity season when the largest weekly value falls below IT_50_, a moderate‐severity season when between IT_50_ and IT_90_, a high‐severity season when between IT_90_ and IT_98_, and a very high‐severity season when above IT_98_.

To assess the validity of using oseltamivir prescriptions among beneficiaries to measure seasonal severity, we compared results from this MEM analysis to analogous results using national surveillance data compiled by the Centers for Disease Control and Prevention (CDC). We used the weighted proportion of ILI among outpatient healthcare providers participating in the US Outpatient Influenza‐like Illness Surveillance Network (ILINet) as the primary reference. As secondary references, we used (i) the proportion of specimens testing positive for influenza virus among those specimens submitted to the World Health Organization Collaborating Laboratories and the National Enteric Virus Surveillance System in the United States (denoted influenza virus data hereafter) for influenza testing and (ii) the Goldstein index, a proxy for the weekly proportion of laboratory‐confirmed influenza infections among those seeking care in the outpatient setting.^15^ These surveillance systems are described elsewhere.^2^ We did not limit surveillance data to people 65 years old and older, as these data are not available stratified by both region and age group. Using the results of the MEM analyses, we used Cohen's weighted κ to measure agreement between the severity of each season as categorized with the CMS data and the severity of each season as categorized with the national surveillance data.^16^ We interpreted Cohen's weighted κ using the Landis‐Koch classification.^17^ As a sensitivity analysis to assess our implicit assumption of a steady population at risk in the CMS data, we also considered the weekly proportion of beneficiaries meeting the case definition (cases per person per week).

We used Spearman's ρ to measure the association between weekly counts in the CMS data and the weekly indicators in the national surveillance data. To assess the relative timing between the CMS data and the national surveillance data, we used time‐lagged cross‐correlations of the time series from the CMS data and national surveillance data.^18^ Briefly, the time‐lagged cross‐correlation measures association between 2 stationary time series, where the time series are shifted in time relative to each other. A positive time lag shifts the second time series back in time relative to the first time series. The time lag with the strongest correlation corresponds to the relative timing of the 2 time series. Because influenza is strongly seasonal, we used a robust seasonal‐trend decomposition to obtain a stationary time series from each data source to compute the time‐lagged cross‐correlations.^19^

For all computations, we used R: A Language and Environment for Computing (version 3.3.1, 2016, R Foundation for Statistical Computing, Vienna, Austria). We used the R packages mem: Moving Epidemic Method R Package (2014, Jose E. Lozano Alonso) and psych: Procedures for Personality and Psychological Research (2017, William Revelle).

## 3 RESULTS

### 3.1 Antiviral prescriptions

**Figure 1:**
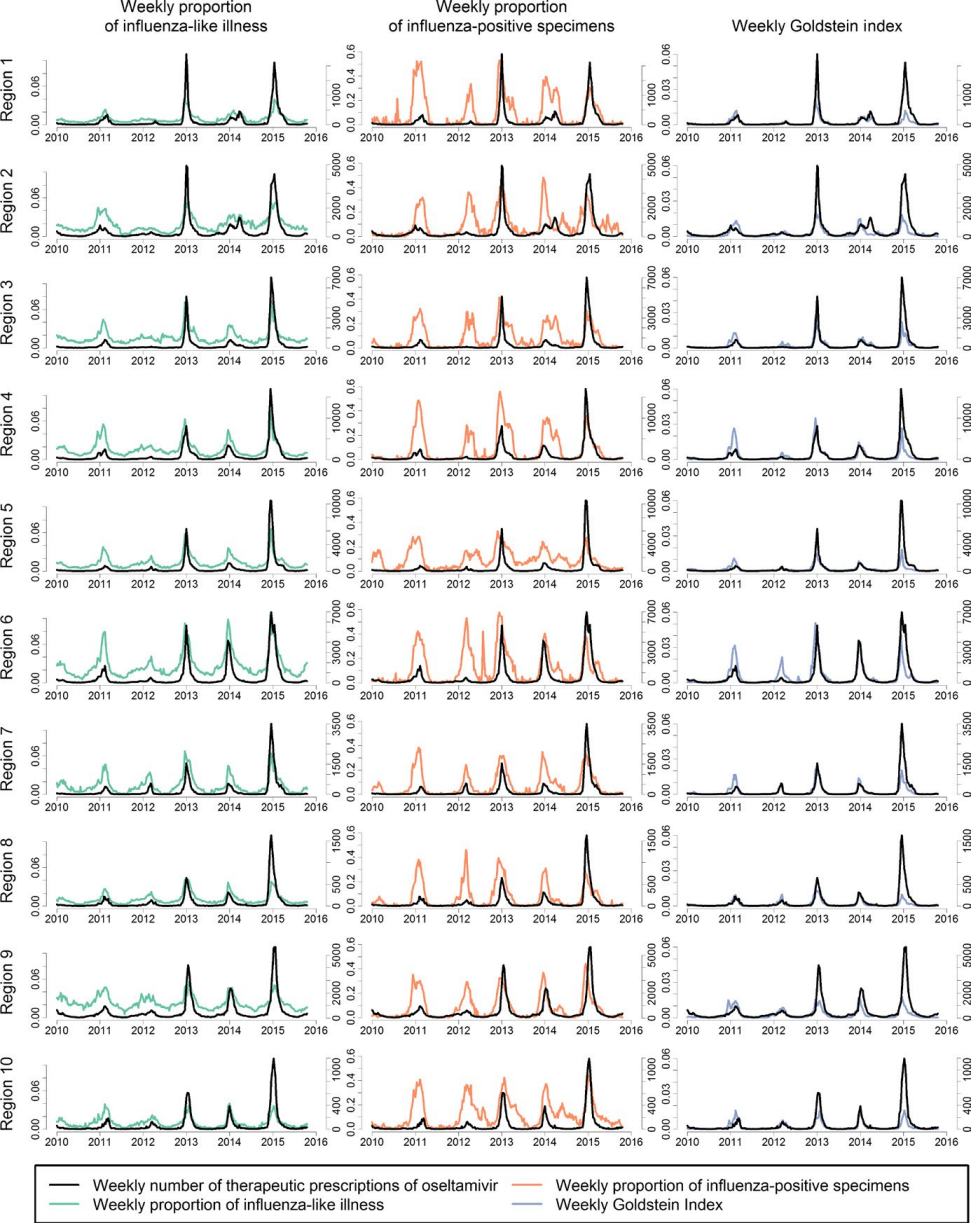
Weekly number of therapeutic oseltamivir prescriptions plotted against indicators for influenza in national surveillance from January 3, 2010, until October 31, 2015

From January 3, 2010, to October 31, 2015, beneficiaries received 1 037 157 therapeutic courses of oseltamivir, including courses preceded by a RIDT and those not associated with a RIDT. The total number varied by administrative region (Table 1). A time series of the number of oseltamivir prescriptions differed in peak magnitude and timing among regions (Figure 1). Stratified by season and region, the peak weekly numbers of oseltamivir prescriptions strongly correlated with the total number of prescriptions (ρ = 0.96).

**Table 1:** Therapeutic oseltamivir prescriptions during the 2010–11 to 2014–15 seasons

Region	US States in Region	Number of AV^a^	Number of AV^a^ following a RIDT^b^	Percent of AV^a^ with a previous RIDT^b^	Average annual number of beneficiaries	Average number of AV^a^ per 100 beneficiaries
Region 1	CT, ME, MA, NH, RI, VT	36 455	9420	25.8	1 619 927	2.25
Region 2	NJ, NY	110 430	17 830	16.1	3 559 046	3.10
Region 3	DE, MD, VA, WV	88 875	30 878	34.7	3 178 352	2.80
Region 4	AL, FL, GA, KY, MS, NC, SC, TN	267 865	135 764	50.7	7 155 626	3.74
Region 5	IL, IN, MI, MN, OH, WI	126 573	37 432	29.6	5 588 909	2.26
Region 6	AR, LA, NM, OK, TX	151 727	75 884	50.0	3 521 609	4.31
Region 7	IA, KS, MO, NE	44 536	22 218	49.9	1 598 942	2.78
Region 8	CO, MT, ND, SD, UT, WY	22 163	8510	38.4	959 550	2.31
Region 9	AZ, CA, HI, NV	137 997	11 722	8.5	4 782 023	2.88
Region 10	AK, ID, OR, WA	18 786	4010	21.3	1 266 535	1.48

a AV = Therapeutic oseltamivir prescriptions.

b RIDT = Rapid influenza diagnostic test.

During the study period, the weekly number of prescriptions within a region correlated with the weekly proportion of ILI from CDC surveillance data, with the weekly proportion of influenza‐positive laboratory specimens, and with the weekly Goldstein index (Table 2, Figure 1). The median difference between the week of the peak number of oseltamivir prescriptions within a region and the week of the peak proportion of ILI from CDC surveillance data was 0 weeks (interquartile range [IQR] = 0.75‐2.00 weeks). The median difference in peak timing with the influenza virus data was 1 week (IQR = −1.00 to 3.00 weeks), and the median difference with the Goldstein index was 0 weeks (IQR = −1.00 to 2.00 weeks).

**Table 2:** Spearman's correlation coefficients between therapeutic prescriptions of oseltamivir and indicators of influenza in national surveillance by region during the 2010‐11 to 2014‐15 seasons

Region	Correlation with AV^a^			Correlation with AV^a^ Following a RIDT^b^		
	ILI^c^	Virus^d^	Goldstein index	ILI^c^	Virus^d^	Goldstein index
Region 1	0.89	0.80	0.84	0.80	0.74	0.78
Region 2	0.80	0.63	0.75	0.78	0.70	0.80
Region 3	0.79	0.69	0.74	0.73	0.74	0.78
Region 4	0.77	0.79	0.82	0.73	0.80	0.83
Region 5	0.93	0.66	0.82	0.87	0.65	0.78
Region 6	0.94	0.71	0.81	0.92	0.71	0.80
Region 7	0.89	0.84	0.91	0.80	0.79	0.84
Region 8	0.87	0.85	0.87	0.78	0.80	0.81
Region 9	0.76	0.81	0.84	0.52	0.73	0.73
Region 10	0.80	0.63	0.78	0.64	0.65	0.72

a AV = Therapeutic oseltamivir prescriptions.

b RIDT = Rapid influenza diagnostic test.

c Proportion of influenza‐like illness among outpatient visits.

d Proportion of influenza‐positive specimens.

Positive time lags indicate changes in the CMS data preceded changes in the national influenza surveillance data. The correlation between the weekly number of prescriptions and the weekly proportion of ILI from CDC surveillance data was strongest for a lag of 1 week for region 3 and region 7, −1 week for region 1, and 0 weeks for the other administrative regions. The regional correlation between the weekly number of oseltamivir prescriptions and the proportion of influenza‐positive specimens was strongest for a median lag time of 2 weeks (IQR = 1.25‐3.75 weeks). The regional correlation between the weekly number of AV and the Goldstein index was strongest for a median lag time of 1 week (IQR = 1.00 week).

Using the weekly number of oseltamivir prescriptions from the 2010‐11 to 2014‐15 seasons, the national IT_50_, IT_90_, and IT_98_ were 11 234 prescriptions per week, 40 209 per week, and 86 605 per week, respectively. The national weekly number of prescriptions exceeded the IT_50_ for 8 weeks during the 2012‐13 season, 3 weeks during the 2013‐14 season, and 12 weeks during the 2014‐15 season. The weekly number of prescriptions exceeded the IT_90_ for 3 weeks during the 2014‐15 season. The weekly number of prescriptions did not exceed the IT_98_ during the study period. Nationally, the MEM method categorized the seasons 2010‐11 and 2011‐12 as low severity; 2012‐13 and 2013‐14 as moderate severity; and 2014‐15 as high severity.

**Figure 2:**
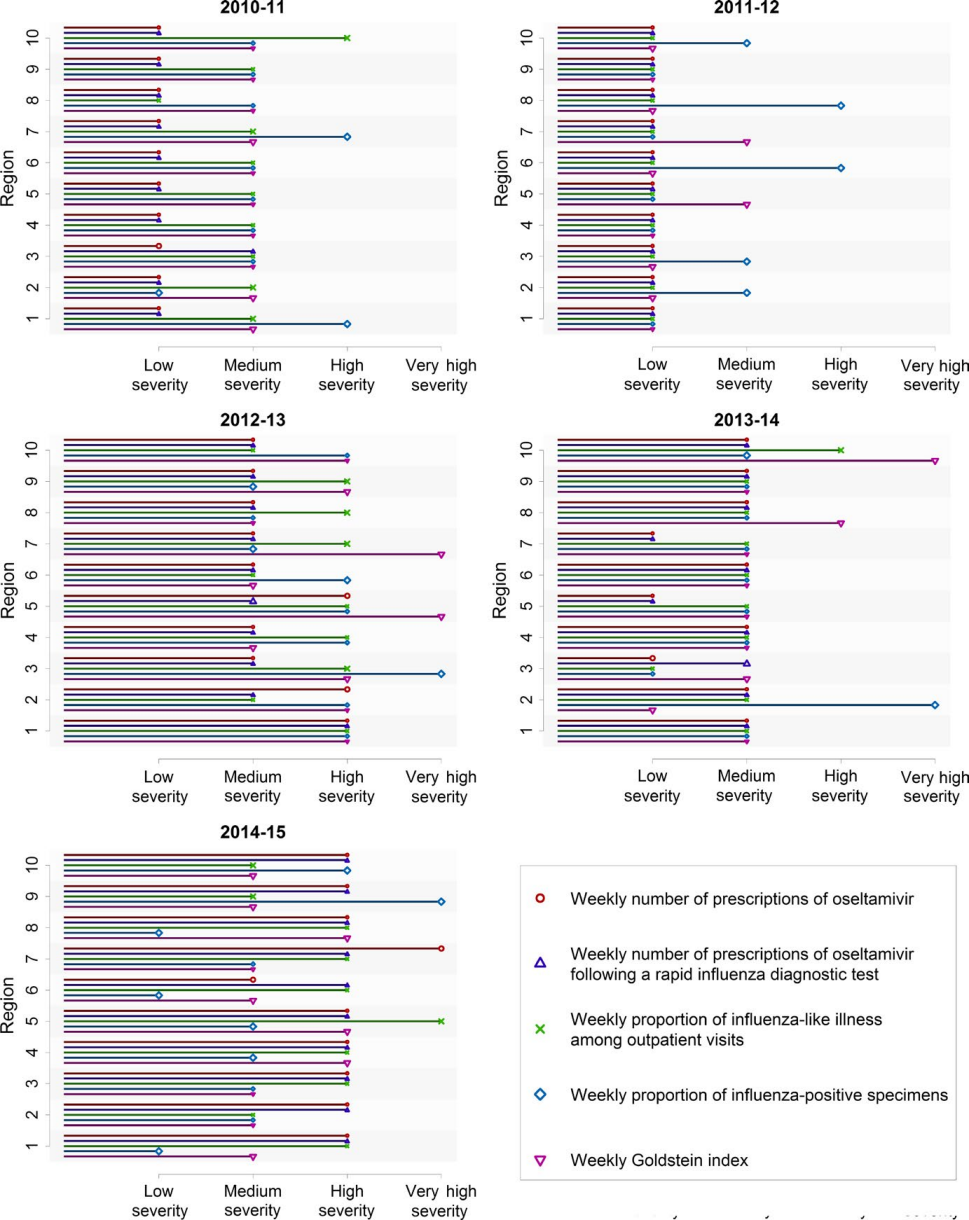
Comparison of seasonal severity of influenza from 2010‐11 to 2014‐15 using the moving epidemic method with different data: weekly number of therapeutic prescriptions of oseltamivir, weekly number of therapeutic prescriptions of oseltamivir following a rapid influenza diagnostic test, the weekly proportion of outpatient visits attributable to an influenza‐like illness, the weekly proportion of influenza‐positive specimens, and the Goldstein index

The ITs for the number of oseltamivir prescriptions varied across regions (Table S1). Agreement between the seasonal severity using the weekly number of prescriptions and the weekly proportion of ILI was substantial (κ = 0.62; Table 3). The 2010‐11 season was categorized as a low‐severity season using the weekly number of prescriptions for all regions, but most regional severities were higher when using national surveillance data (Figure 2).

**Table 3:** Cohen's weighted κ measuring agreement between seasonal severity in the medical claims data and seasonal severity in the national surveillance data from the 2010‐11 through 2014‐15 influenza season

Prescriptions of oseltamivir	Weekly proportion of outpatient influenza‐like illness	Weekly proportion of influenza‐positive specimens	Weekly Goldstein index
Per wk			
AV^a^	0.62	0.23	0.46
AV^a^ after RIDT^b^	0.62	0.13	0.41
Per person per wk			
AV^a^	0.66	0.28	0.50
AV^a^ after RIDT^b^	0.66	0.16	0.41

a AV = Therapeutic prescriptions of oseltamivir.

b RIDT = Rapid influenza diagnostic test.

### 3.2 Oseltamivir dispensed following a RIDT

**Figure 3:**
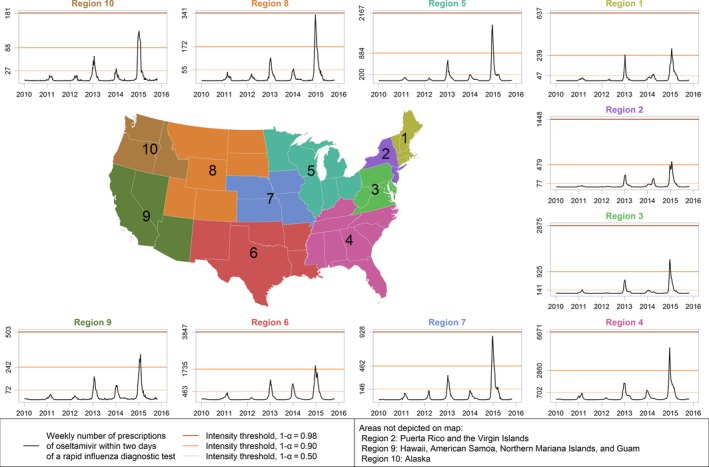
The weekly number of therapeutic oseltamivir prescriptions within 2 d of a rapid influenza diagnostic test in each Department of Health and Human Services Region

**Figure 4:**
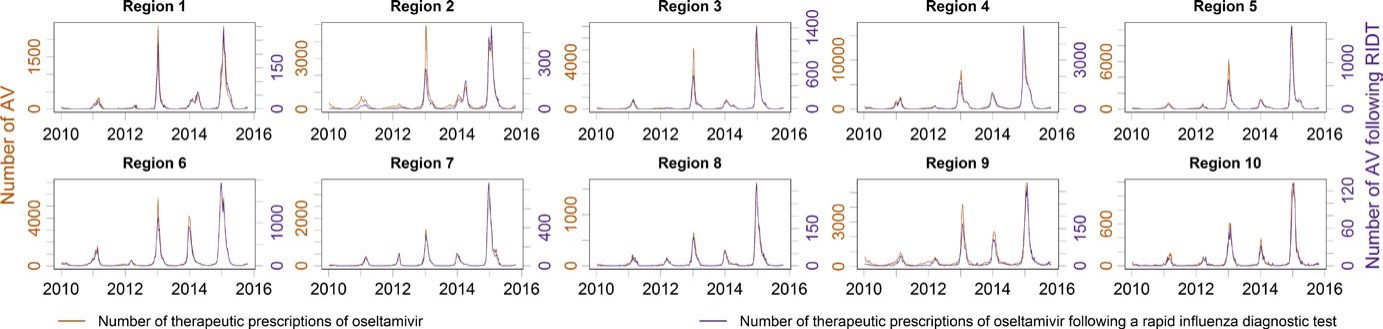
The weekly number of therapeutic oseltamivir prescriptions plotted with the weekly number of prescriptions following a rapid influenza diagnostic test from January 3, 2010, until October 31, 2015

From January 3, 2010, to October 31, 2015, beneficiaries received 353 668 courses of oseltamivir within 2 days of a RIDT: 66% of courses of oseltamivir were not associated with a RIDT within 2 days. The total number of prescriptions dispensed following a RIDT varied by region (Table 1). A time series of the number of prescriptions following a RIDT differed in peak magnitude and timing among regions (Figure 3). Other than scale, regional trends in the number of prescriptions were similar to trends in the number of prescriptions following a RIDT (Figure 4). Stratified by season and region, the peak weekly number of oseltamivir prescriptions following a RIDT strongly correlated with the total number of prescriptions following a RIDT (ρ = 0.95).

**Figure 5:**
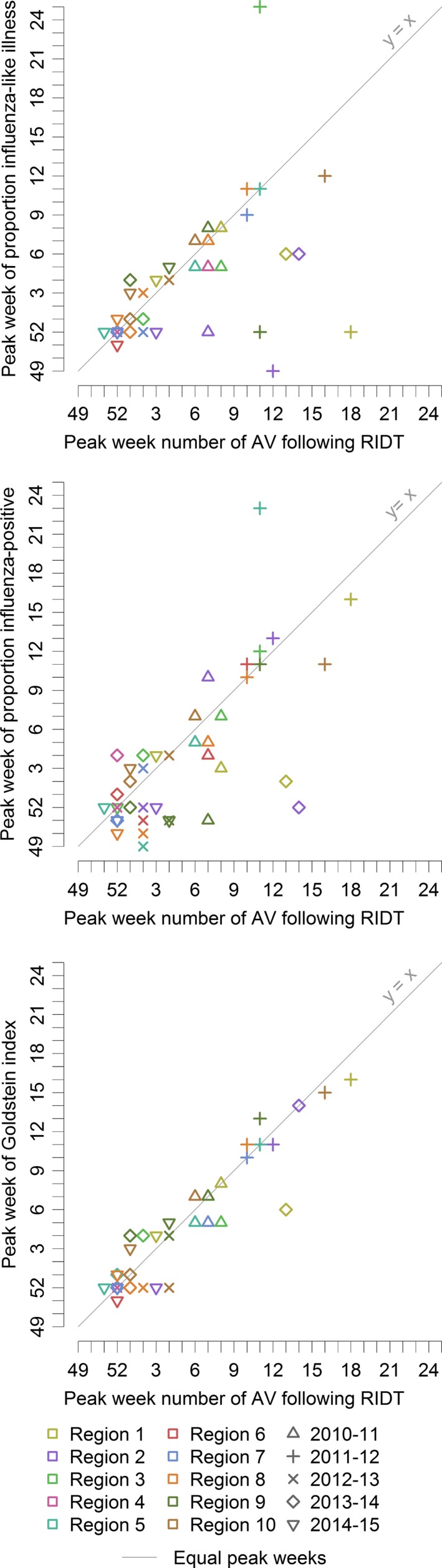
The peak week of the number of therapeutic oseltamivir prescriptions following a rapid influenza diagnostic test compared to the peak week of indicators of influenza in national surveillance for the 2010‐11 through 2014‐15 seasons

During the study period, the number of prescriptions following a RIDT within a region correlated with the proportion of outpatient visits for ILI, with the proportion of influenza‐positive laboratory specimens, and with the Goldstein index (Table 2). The median difference between the week with the largest number of prescriptions following a RIDT within a region and the largest proportion of ILI was 0 weeks (IQR = −1.00 to 2.00 weeks) (Figure 5). The median difference in peak timing with the influenza virus data was 1 week (IQR = −1.00 to 2.75 weeks) and with the Goldstein index was 0 weeks (IQR = −0.75 to 2.00 weeks) (Figure 5).

Positive time lags indicate changes in the CMS data preceded changes in the national influenza surveillance data. The correlation between the weekly number of prescriptions following a RIDT and ILI was strongest with a lag of 1 week for region 1; the time lag was 0 weeks for the other regions. The regional correlation between the weekly number of prescriptions following a RIDT and the proportion of influenza‐positive specimens was strongest for a median lag time of 3 weeks (IQR = 0.50‐3.75 weeks). The regional correlation between the weekly number of prescriptions following a RIDT and the Goldstein index was strongest for a median lag time of 0.5 weeks (IQR = 0.00‐1.00 weeks).

Using the weekly number of oseltamivir prescriptions following a RIDT from 2010‐11 through 2014‐15, the national IT_50_, IT_90_, and IT_98_ from the MEM analyses were 1921 prescriptions following a RIDT per week, 7452 per week, and 16 866 per week, respectively. The national weekly number of prescriptions following a RIDT exceeded IT_50_ for 7 weeks in 2012‐13, 4 weeks in 2013‐14, and 15 weeks in 2014‐15; the weekly number exceeded IT_90_ for 3 weeks in 2014‐15. The weekly number of prescriptions following a RIDT did not exceed any of the IT_98_ for any region during the study period. Nationally, the MEM method categorized the seasons 2010‐11 and 2011‐12 as low severity; 2012‐13 and 2013‐14 as moderate severity; and 2014‐15 as high severity.

Within regions, ITs for the weekly number of oseltamivir prescriptions following a RIDT varied (Table S1). Agreement between seasonal severity using the weekly number of prescriptions and seasonal severity using the weekly proportion of ILI was substantial (κ = 0.62; Table 3). When the CMS data and data from another surveillance system exceeded an IT, the time difference was within 3 weeks 85% of the time. Outliers to this trend included a late peak in the oseltamivir data for region 1 and region 2 during the 2013‐14 season relative to influenza virus data (Figure 5).

### 3.3 Comparison of oseltamivir prescriptions with oseltamivir prescriptions following RIDT

**Figure 6:**
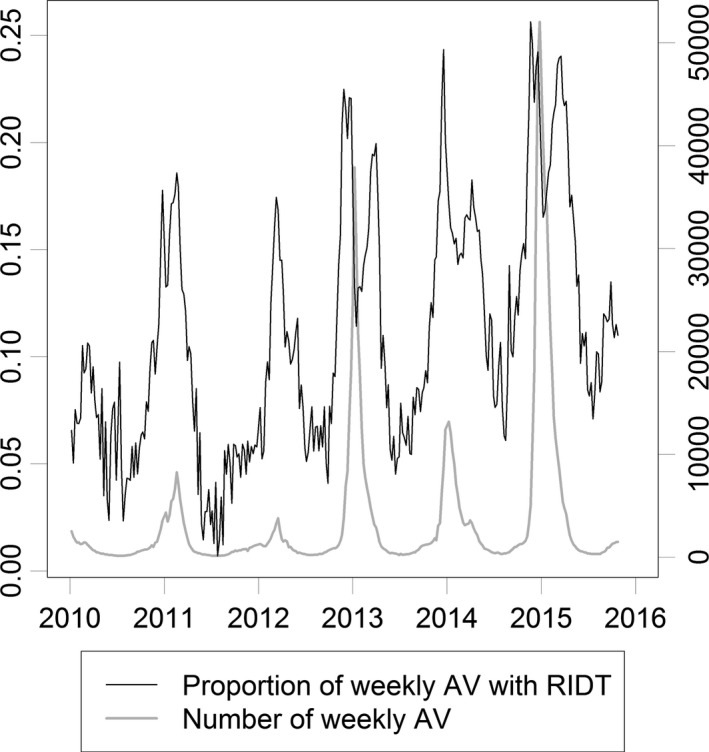
The national weekly number of therapeutic oseltamivir prescriptions (AV) and the proportion of prescriptions preceded by a rapid influenza diagnostic test (RIDT)

The national weekly number of oseltamivir prescriptions strongly correlated with the weekly number of prescriptions following a RIDT (ρ = 0.98). Similarly, the regional weekly number of prescriptions strongly correlated with the weekly number of prescriptions following a RIDT (median ρ = 0.90, IQR = 0.87‐0.93). The proportion of oseltamivir prescriptions following a RIDT among those receiving prescriptions was seasonal (Figure 6) and varied by region (Table 1).

## 4 DISCUSSION

The weekly number of oseltamivir prescriptions dispensed to Medicare beneficiaries 65 years old and older within 2 days of a RIDT strongly correlated with clinical and influenza virus data for all ages from existing national surveillance systems for influenza (Table 2). We found no evidence of a meaningful time lag between prescriptions following a RIDT with outpatient ILI, indicating that the 2 sources of data yield similar conclusions about the timing of influenza activity. At the national level, our indicators of influenza assigned the same seasonal severity categories as previous work with analogous methods using national surveillance data for people 65 years old and older.^20^ Our results suggest that oseltamivir prescriptions following a RIDT may serve as a proxy for laboratory‐confirmed influenza in epidemiological studies using medical claims in the United States.

The peak week of the number of oseltamivir prescriptions following a RIDT was often different from the peak week of ILI, especially for the 2011‐12 season (Figure 5). We did not find as many differences when we compared the weekly number of prescriptions following a RIDT with the Goldstein index, suggesting these differences in peak timing may be partly attributable to ILIs with a non‐influenza etiology (Figure 5). The late peaks of prescriptions following a RIDT during the 2013‐14 season in the Northeast (regions 1 and 2) may be attributable to a second wave of influenza (Figure 5). The first wave was predominantly influenza A (H1).^21^ The second wave consisted of both influenza A (H3) and influenza B, and this wave was more prominent in people 65 years old and older.^22^ Also, the second wave was more prominent in the Northeast relative to the other US regions.^22^ Given this context, these late peaks suggest our indicator of influenza is sensitive to incidence of influenza among people 65 years old and older.

Our findings are subject to several limitations. The medical claims data from CMS are representative for community‐level healthcare visits among people 65 years old and older, who account for the majority of hospitalizations and deaths attributable to influenza in the United States.^7^, ^23^ However, Medicare data do not capture influenza infections in younger persons which limits the representativeness of our results. We highlight 2 results illustrating this limitation. First, the 2014‐15 influenza season was especially severe for people 65 years old and older, which may explain why the severity of this season was categorized higher when using the CMS medical claims data than when using influenza virus data from surveillance data for most regions (Figure 2).^24^ Next, a second wave of influenza during the 2012‐13 season was more prominent in the influenza virus data than in the CMS medical claims data (Figure 1). Nationally, influenza B among school‐aged children was largely responsible for this second wave in the influenza virus data (FluView Interactive, https://gis.cdc.gov/grasp/fluview/flu_by_age_virus.html). The different population age structures between the CMS medical claims data and the influenza virus data may explain the relatively long time lag between these 2 data sources. Additionally, our results are limited to the community setting, as the medical claims data do not specify drugs administered during inpatient care. Conversely, influenza illnesses that do not come to medical attention are also absent from medical claims data. Therefore, the pathogenicity and virulence of circulating influenza viruses—which may differ between people 65 years old and older and younger people—affect our indicator in complex ways.^25^, ^26^

Results from the MEM analyses of medical claims data agreed substantially with results from ILINet; however, they differed with respect to results from the influenza virus data (Table 3). Agreement in seasonal severity was poor in the 2010‐11 season (Figure 2), a season that nationally was less severe for people 65 years old and older relative to younger people.^20^ In general, using ILI as a case definition may have low predictive value for influenza among young children because of high incidence of respiratory syncytial virus, rhinovirus, and metapneumovirus infections in this age group, especially when influenza is not circulating widely.^27^, ^28^, ^29^ This disagreement in assessment of seasonal severity may be attributed to the discrepancy in source populations, suggesting our indicators are specific to influenza activity in people 65 years old and older.

The use of RIDT and oseltamivir in community settings varies within and across seasons.^11^, ^30^ Reliance on RIDT in the community setting may decrease if real‐time reverse transcription polymerase chain reaction assays for influenza become more prevalent. Changes in care‐seeking behavior and clinical practice will influence the proportion of influenza cases meeting our definition in these medical claims data. Limiting the data to oseltamivir prescriptions following a RIDT did not qualitatively alter our findings. At a national level, the proportion of prescriptions following a RIDT tracked the seasonal trend of influenza (Figure 6), suggesting healthcare providers generally test when the positive predictive value is highest. As 66% of prescriptions were not associated with a RIDT within 2 days, our data suggest clinicians treat suspected influenza infections among Medicare beneficiaries empirically. While oseltamivir prescriptions in our data represent treatment of suspected or confirmed influenza A or B in a community setting, interpretation of prescriptions following a RIDT must also consider how clinicians use RIDT, which may vary season to season.^31^, ^32^, ^33^, ^34^

We believe medical claims data for prescriptions of oseltamivir show potential to monitor the timing and severity of seasonal influenza activity in the United States. Although these data are not timely enough for real‐time surveillance, this may change if the use of electronic billing continues to increase. Next, we plan to assess the usefulness of medical claims data on a metropolitan level to investigate spatiotemporal trends in influenza activity at a finer resolution, as current US influenza surveillance data are aggregated to a state or multistate level. Because the volume of prescriptions is much larger when not limited to those following a RIDT, we plan to use the weekly number of oseltamivir prescriptions as an indicator of influenza activity when analyzing claims data on metropolitan areas.

## DISCLAIMER

The findings and conclusions in this report are those of the authors and do not necessarily represent the official position of the Centers for Disease Control and Prevention or the Food and Drug Administration.
