# Supplementary material for: Evaluating oseltamivir prescriptions in Centers for Medicare and Medicaid Services medical claims records as an indicator of seasonal influenza in the United States
Source: Influenza Other Respir Viruses. 2018 Mar 25;12(4):465–74. doi: 10.1111/irv.12552 (PMC6005588; doi:10.1111/irv.12552)
Supplement: Supplementary file 1 [file IRV-12-465-s001.docx]

SUPPLEMENTAL TABLES

## Table S1: Intensity thresholds for the weekly number of therapeutic oseltamivir prescriptions from the moving epidemic analyses of the 2010–11 through 2014–15 influenza seasons.

| Region | Intensity Thresholds for AV^1^ | | |  | Intensity Thresholds for AV^1^ Following a RIDT^2^ | | |
| --- | --- | --- | --- | --- | --- | --- | --- |
|  | IT_50_ | IT_90_ | IT_98_ |  | IT_50_ | IT_90_ | IT_98_ |
| Region 1 | 412 | 1,869 | 4,649 |  | 47 | 239 | 637 |
| Region 2 | 1,105 | 4,187 | 9,341 |  | 77 | 479 | 1,448 |
| Region 3 | 877 | 5,251 | 15,438 |  | 141 | 925 | 2,875 |
| Region 4 | 2,830 | 10,805 | 24,224 |  | 702 | 2,860 | 6,671 |
| Region 5 | 1,428 | 6,061 | 14,485 |  | 200 | 884 | 2,167 |
| Region 6 | 1,932 | 7,004 | 15,216 |  | 463 | 1,735 | 3,847 |
| Region 7 | 643 | 1,850 | 3,498 |  | 146 | 462 | 9,28 |
| Region 8 | 292 | 904 | 1,790 |  | 55 | 172 | 341 |
| Region 9 | 1,648 | 4,977 | 9,686 |  | 72 | 242 | 503 |
| Region 10 | 265 | 825 | 1,636 |  | 27 | 88 | 181 |

^1^AV = therapeutic oseltamivir prescriptions

^2^RIDT = rapid influenza diagnostic test
